# Removing a bent femoral nail - man versus metal: A case report

**DOI:** 10.1016/j.ijscr.2022.107679

**Published:** 2022-09-19

**Authors:** Marlon M. Mencia, Reena Moonsie

**Affiliations:** a Bungalow 5, Department of Clinical Surgical Sciences, University of the West Indies, Port of Spain General Hospital, Port of Spain, Trinidad, West Indies, Trinidad and Tobago; b Eric Williams Medical Sciences Complex, University of the West Indies, Champ Fleur, Trinidad, West Indies, Trinidad and Tobago

**Keywords:** Case report, Intramedullary, Femoral nail, Bent, Hacksaw, Low-resource

## Abstract

**Introduction:**

Intramedullary nailing is the treatment of choice for femoral shaft fractures in adults with excellent clinical results and low complication rates reported in the literature. However, in situ bending of a femoral nail is a rare complication that merits special attention. While there are several extraction techniques and algorithms the scientific evidence to support these decision-making tools is unconvincing.

**Presentation of case:**

A 26-year old man presented to the Accident and Emergency Department with a deformed thigh following a low-energy injury. Radiographs showed a bent femoral nail in situ and the patient disclosed that he had surgery four weeks earlier for a fractured femur sustained in a motor vehicle accident. A treatment algorithm was followed in planning the surgical strategy, but ultimately a simple hacksaw blade was used to cut and remove the nail. The fracture which was stabilised by exchange nailing went on to uncomplicated union and the patient recovered fully.

**Discussion:**

Non-invasive methods of removing a bent femoral nail are often unsuccessful and may result in iatrogenic injuries. Surgeons should assess the available local resources and first consider using simple open methods when attempting to remove a bent femoral nail.

**Conclusion:**

Open extraction methods often disregard the low-resource environment in which many surgeons work. We describe a simple and economical technique that uses a regular hacksaw blade to cut and remove a bent femoral nail.

## Highlights

• Using modern implants, insitu bending of a femoral nail is a rare occurrence.
• Closed extraction methods are often unsuccessful and may lead to iatrogenic injuries.
• An open technique using a simple hacksaw can be used in low-resource settings without access to sophisticated extraction tools.

## 1 Introduction

It is widely accepted that reamed intramedullary nailing is the gold standard of care for managing femoral shaft fractures [1], [2]. As internal splints, intramedullary nails are load-sharing devices that facilitate early weight-bearing and accelerated rehabilitation [3], [4]. A study by Brumback et al. demonstrated that without bony contact, a statically locked 12 mm diameter femoral nail could easily withstand the typical forces of early weight-bearing [5]. However, fatigue failure and nail breakage may occur if the weight-bearing forces exceed the strength of the nail. Using modern surgical techniques and nails complications caused by implant failure are rare [2], [6].

One curious complication that occurs infrequently is bending of the nail secondary to additional trauma. In 2020, Dunleavy et al. conducted a systematic review of the literature but could identify only 27 cases of femoral nail bending [7]. More recently, two additional cases were reported by Suh et al. [8]. Invariably, the bent femoral nail needs to be removed. While several different techniques have been used for nail extraction, no one method has gained universal acceptance [8], [9], [10], [11], [12], [13], [14]. Developing countries face additional obstacles as specialized instruments such as diamond-tipped drills or high-speed burrs that simplify nail removal are not readily available. In managing these challenging cases, surgeons frequently have to improvise, repurposing industrial tools for use in the operating theatre.

We report a case of a 26-year old man who presented with a bent femoral nail in situ, which was successfully removed using a conventional hacksaw blade. This work has been reported in line with the SCARE 2020 criteria [15].

## 2 Presentation of case

**Fig. 1:**
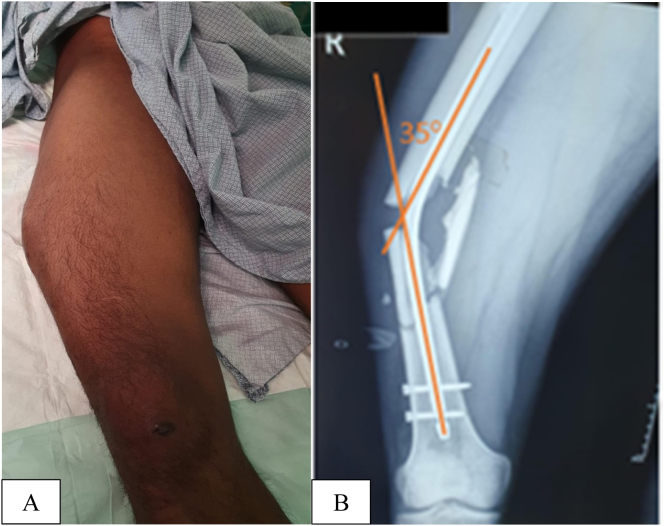
A. Clinical photograph of the patient's thigh showing a significant varus deformity. B. Anteroposterior radiograph of the femur illustrating the bent intramedullary femoral nail in-situ.

A 26-year-old man presented to the Emergency Department with an obvious deformity of his right thigh following a fall Fig. 1A. He revealed that four weeks prior to this admission, he fractured his femur in a motor vehicle accident and underwent femoral nailing at a nearby hospital.

Despite the striking limb asymmetry, we were surprised to find that he was remarkably comfortable. Initial radiographs showed a comminuted midshaft femoral fracture with a 35° varus-angulated intramedullary nail Fig. 1B. We informed the patient that he would require revision surgery and prepared him for the next trauma list.

Under a general anaesthetic, we tried to straighten the nail using the technique described by Patterson et al., but after several unsuccessful attempts, we abandoned this method [16]. We then tried to remove the nail without straightening it. After removing the locking bolts, we used the original hip incision to access the top of the nail and attached the extraction device. Using the slap-hammer, we laboured unsuccessfully to back the nail out. As a final resort, we decided to open the fracture site and either weaken and straighten the nail or completely transect it.

Using a direct lateral approach, we made a 10 cm incision directly over the apex of the deformity and worked our way to the fracture site. The gleaming metallic surface of the nail was easily seen in the depths of the wound. In anticipating the need to cut the nail, we had earlier sterilised two new metal-cutting hacksaw blades. The blades were acquired from the hospital maintenance department and are part of their standard inventory. Two Hohmann retractors placed on either side of the femur opened the surgical field, allowing us to use the hacksaw freely. We covered the surrounding soft tissues with a moist sponge to collect any metal debris produced while cutting the nail.

**Fig. 2:**
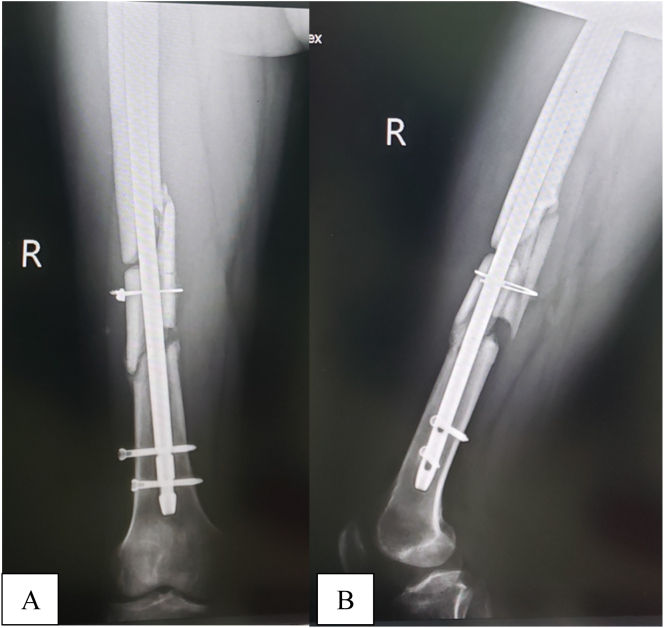
Immediate postoperative radiographs of the femur. Anteroposterior (A) and lateral (B) views showing a locked intramedullary femoral nail. Note the cerclage cable stabilising the segmental fracture.

To our surprise, we discovered that the nail was relatively easy to cut, and in a short time, a 5 mm lateral-based wedge of metal was removed. As we then attempted to straighten the nail, it suddenly snapped in two. Fortunately for us, this made it easy to remove the nail; the proximal portion was extracted via the trochanteric entry point and the distal portion through the fracture site. With the nail removed, we noticed that there was now a bivalved middle segment of bone. We used cerclage cable to approximate the middle segment of the femoral shaft before proceeding with exchange nailing Fig. 2. The femoral canal was carefully reamed to avoid “spinning” of the middle segment, and a 12 mm locked intramedullary nail was introduced to stabilize the fracture. The operation time was 136 mins, and the estimated blood loss was 350 mls. The patient's postoperative course was uneventful, and he was discharged three days after surgery.

**Fig. 3:**
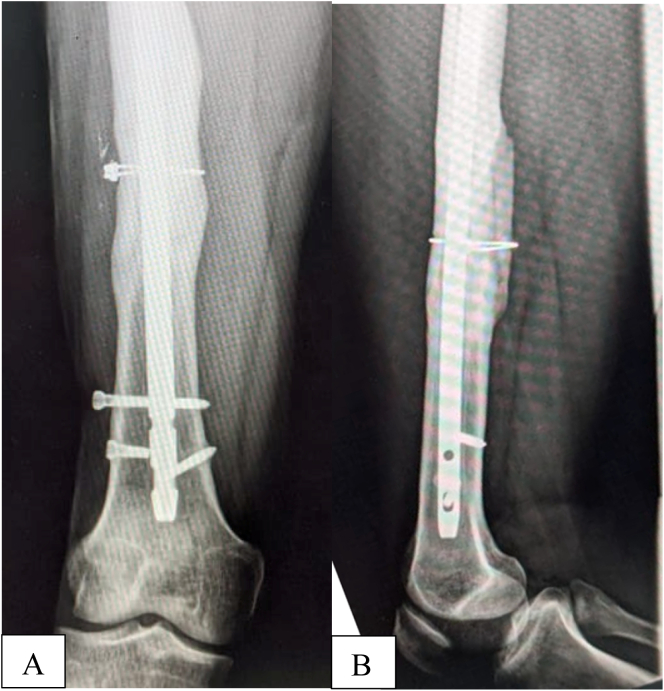
Anteroposterior (A) and lateral radiograph (B) of the femur two years after exchange nailing illustrating complete healing of the femoral fracture and extensive bone remodelling. Note: self-dynamization of the nail through the broken distal locking bolt and the missed proximal locking bolt.

The patient's incisions healed without any evidence of infections and he progressed well under supervised physiotherapy. One year after exchange femoral nailing, the patient has returned to work as a fulltime salesman. His gait is normal with excellent knee movement (Range 0° - 135°) and he is very satisfied with his level of function (Health-related quality of life (EQ-5D [EuroQoL 5 Dimensions] index score: 1 and EQ-VAS[visual analogue score]: 95)) [17]. Radiographs show union of the fracture Fig. 3.

The patient gave informed consent for the publication of this case report and the use of his anonymized images.

## 3 Discussion

Removing an intramedullary nail can be a technically demanding surgical problem. Several variables contribute to the task complexity, including in low-resource centres the lack of specific metal-cutting tools. Since no method has emerged as superior to the others, surgeons must look to the literature for guidance.

**Fig. 4:**
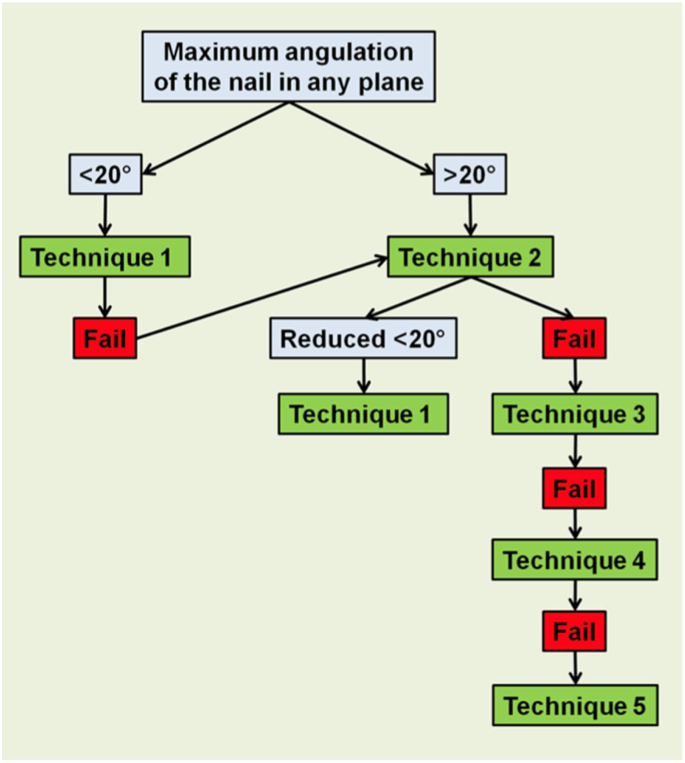
Algorithm for removal of bent intramedullary nails. Reprinted with permission from Springer Nature Customer Service Centre GmbH: Springer [Archives of Orthopaedic and Trauma Surgery] (Removal of a bent intramedullary nail in lower extremity: report of two cases and review of removal techniques, Ozkan Klose et al.) [CC-BY-NC-SA] (2015).

A systematic review of the literature is often used to create clinical practice guidelines (CPGs) which are then presented with the help of an algorithm. Our literature search found only two systematic reviews on the management of a bent femoral nail [7], [9]. Based on their evaluation, Kose et al. proposed an algorithm for the removal of a bent intramedullary nail [9] Fig. 4. However critical analysis of the studies on which these recommendations are founded reveal that there were no patients with angular deformity <20°. Furthermore, only one patient with a deformity >20° was successfully treated by straightening and extirpation of the nail. If we make the reasonable assumption that most surgeons will attempt a closed manipulation of the nail before proceeding further, then 93 % (25/27) of patients required a more complex technique to remove the femoral nail. On the surface a closed manipulation might appear to be a benign procedure, however there is risk to soft tissues in particular the collateral ligaments of the knee and iatrogenic fractures have been reported [18] Undiscerning adherence to guidelines may result in ineffective or harmful practices. We are in agreement with Kose et al. who argue that angular deformity is only one factor which can influence the ease with which a nail can be removed, several other factors including the type and size of femoral nail and the degree of comminution have a significant role [9]. In the end, the complexity of clinical decision making cannot be reduced to a simplistic algorithm of binary choices [19].

Several techniques for cutting an intramedullary nail have been described but to date there is no universally accepted method. In 2020 Dunleavy et al. conducted the largest study on the surgical management of angulated femoral intramedullary nails [7]. Twenty-five studies (27 patients) were included in their analysis, which revealed that in 65 % of cases either a metal cutting burr or drill bit was used to section the implant. Specialized high speed steel (HSS) drill bits made of titanium or tungsten carbide burrs are required to cut metal. In wealthy countries with a modern healthcare system these instruments are readily accessible. However in resource poor countries, surgeons must use their ingenuity and resourcefulness to manage these situations. An example of this comes in one study from Malaysia and two from India in which a jumbo cutter was used to sever a femoral nail [10], [11], [12]. In another paper from Cambodia, Odendaal et al. attached an industrial diamond-tipped cutting disk purchased from the local hardware to an angle grinder to successfully cut a femoral nail [13]. Similar to the technique described by Nicolaides et al. we used a hand-powered metal cutting saw (hacksaw) to transect the femoral nail [14] Table 1. summarises the techniques used to cut a femoral nail during extraction. Our method to remove a bent femoral nail is simple despite being labour-intensive. It's relevance to low-resource settings resides in the use of an ordinary hacksaw which can be found in the maintenance department of any hospital. Additionally there is minimal risk of soft tissue injury and less scatter of the metal filings which facilitates it removal from the surgical field.

**Table 1:** Summary of demographic data and transection techniques used to remove a femoral nail from previous studies.

Author (year)	Age/sex	Deformity	Type of nail	Method of transection	Definitive treatment	Postoperative course
Bielejeski and Garrick [20] (1970)	NR	30° valgus	Kuntscher SS	Dental cutting disc -motorised	NR	NR
LaSalle and Horwitz [21] (1974)	12/M	30° anterolateral	Schneider SS	Dental drill and disc-mandrel unit	Kuntscher Nail and autogenous iliac bone graft	Uneventful
Nicholson et al. [22] (1998)	18/M	42° varus	Grosse and Kempf SS	Diamond cutting burr and Midas Rex	Proximally locked femoral nail	Uneventful
Kockesen et al. [23](2002)	37/M	42° varus	Kuntscher SS	Metal cutting saw- manual	Intramedullary locked nail	Union at 4 months
Nicolaides et al. [14] (2004)	20/M	85° varus	SS	Metal cutting blade	Intramedullary locked nail	Union at 9 months
Singh et al. [10] (2004)	45/M	35° varus	Kuntscher SS	Jumbo Pin Cutter	NR	NR
Stahel et al. [24] (2009)	42/M	60° apex anterior	Schneider SS	Metal cutting circular saw	NR	NR
Bicici et al. [25] (2013)	35/M	23° varus	SS	Diamond cutting bur and Midas Rex	NR	NR
Dhanda et al. [11] (2015)	26/M	42° varus	Locked Nail	Jumbo Cutter	Statically locked femoral nail and autogenous iliac bone graft	Uneventful
Odendaal et al. [13] (2016)	43/M	20° varus	Kuntscher SS	Diamond tip cutting disc	Intramedullary Kuntscher nail and autogenous iliac bone graft	Uneventful
Canton et al. [26] (2019)	19/M	35° varus	T2 Stryker Ti	Diamond Burrs	T2 Recon Stryker and distal femoral locking plate	Union at 10 months
Sa'aid et al. [12] (2021)	17/F	30° varus 30° apex anterior	Locked Nail	Jumbo Cutter	Locking plate and bone graft	Uneventful

NR- Not recorded; SS- Stainless Steel; Ti- Titanium.

## 4 Conclusion

The existing literature on removal of a bent intramedullary femoral nail encompasses several different techniques. While no one method has emerged as superior to the others, we caution against simply following a step-wise algorithmic progression of extraction methods. The majority of the world's population live in low-income countries where sophisticated extraction tools are not available. Under these circumstances we suggest that surgeons consider the use of a regular metal-cutting hacksaw blade to section the nail before removal.

## Sources of funding

None.

## Ethical approval

N/A.

## Consent

Written informed consent was obtained from the patient for publication of this case report and accompanying images. A copy of the written consent is available for review by the Editor-in-Chief of this journal on request.

## Guarantor

Marlon M. Mencia

## Provenance and peer review

Not commissioned, externally peer-reviewed.

## CRediT authorship contribution statement

Marlon M. Mencia revised the first draft and wrote final manuscript.

Reena Moonsie collected the data, conceptualised the case report, and wrote the first draft.

Both authors read and approved the final manuscript.

## Declaration of competing interest

None declared.
